# Accessibility of the histone H3 tail in the nucleosome for binding of paired readers

**DOI:** 10.1038/s41467-017-01598-x

**Published:** 2017-11-14

**Authors:** Jovylyn Gatchalian, Xiaodong Wang, Jinzen Ikebe, Khan L. Cox, Adam H. Tencer, Yi Zhang, Nathaniel L. Burge, Luo Di, Matthew D. Gibson, Catherine A. Musselman, Michael G. Poirier, Hidetoshi Kono, Jeffrey J. Hayes, Tatiana G. Kutateladze

**Affiliations:** 1 Department of Pharmacology, University of Colorado School of Medicine, Aurora, CO 80045 USA; 2 Department of Biochemistry and Biophysics, University of Rochester Medical Center, Rochester, NY 14642 USA; 3 Molecular Modeling and Simulation Group, National Institutes for Quantum and Radiological Science and Technology, Kizugawa, Kyoto, 619 0215 Japan; 4 Department of Physics, Ohio State University, Columbus, OH, 43210 USA; 5 Department of Biochemistry, University of Iowa College of Medicine, Iowa City, IA 52242 USA; 6 Present Address: Artificial Intelligence Research Center, Advanced Industrial Science and Technology, Tokyo, 135-0064 Japan

## Abstract

Combinatorial polyvalent contacts of histone-binding domains or readers commonly mediate localization and activities of chromatin-associated proteins. A pair of readers, the PHD fingers of the protein CHD4, has been shown to bivalently recognize histone H3 tails. Here we describe a mechanism by which these linked but independent readers bind to the intact nucleosome core particle (NCP). Comprehensive NMR, chemical reactivity, molecular dynamics, and fluorescence analyses point to the critical roles of intra-nucleosomal histone-DNA interactions that reduce the accessibility of H3 tails in NCP, the nucleosomal DNA, and the linker between readers in modulating nucleosome- and/or histone-binding activities of the readers. We show that the second PHD finger of CHD4 initiates recruitment to the nucleosome, however both PHDs are required to alter the NCP dynamics. Our findings reveal a distinctive regulatory mechanism for the association of paired readers with the nucleosome that provides an intricate balance between cooperative and individual activities of the readers.

## Abstract (web summary)

The chromatin remodeller CHD4 contains two PHD finger reader domains that have been shown to bivalently recognize H3 histone tails. Here, the authors describe a mechanism by which the PHD fingers bind to the intact nucleosome core particle, revealing both cooperative and individual interactions.

## Introduction

The human genome is packed inside the nucleus of cells in the form of chromatin, a highly dynamic complex of DNA and histones. Chromatin undergoes extensive changes during the cell cycle and is required to be relaxed or condensed for the cell to execute vital biological programs such as gene transcription and DNA repair, replication, and recombination. Chromatin restructuring is facilitated by remodeling motors that hydrolyze ATP and utilize the released energy to remove, exchange, or reposition nucleosomes, the primary subunits of chromatin^1^. Many remodeling motors operate as large multiprotein complexes, consisting of regulatory and accessory components organized around a catalytic subunit. Among the major human chromatin-remodeling machines is the nucleosome remodeling and deacetylase (NuRD) complex^2^. It contains the catalytic ATPase/helicase subunit CHD4 (chromodomain, helicase, DNA-binding protein 4), the second catalytic subunit histone deacetylase HDAC1/2, and several accessory, scaffolding and regulatory components^3–5^.

The essential role of the NuRD complex in a wide range of nuclear events, including transcriptional repression, formation of heterochromatin, stem cell maintenance and differentiation, cell cycle progression, and genomic stability has been well documented^6–8^. Deregulation of any of the NuRD subunits is detrimental to the overall function and accumulation of the complex, and is linked to cancer and developmental and neurological disorders^3, 6, 9^. The genomic localization and repressive activity of the NuRD complex rely on the ability of its subunits to interact with DNA, chromatin-associated transcription factors, and histones. The tandem PHD fingers of the CHD4 subunit have been shown to concurrently bind two histone H3 tails, helping to direct or stabilize the complex at specific genomic loci^10–12^. The bivalent engagement of the CHD4 PHD fingers is required for the repressive activity of NuRD, and leads to the displacement of HP1γ from pericentric sites and dispersion of the pericentric heterochromatin mark H3K9me3^10^. In addition, binding of the PHD fingers to histone H3 stimulates the ATPase activity and chromatin-remodeling function of CHD4 and can in turn be augmented by the ATPase domain^13, 14^.

In vivo studies with mutated CHD4 and native chromatin established the importance of histone recognition by both CHD4 PHD fingers in biological functions of the CHD4–NuRD complex, and in vitro binding and structural analyses delineated interfaces between PHDs and histone peptides. Arguably, characterization of interactions with histone peptides has been one of the most straightforward and cost-effective approaches to explore effector–histone complexes. While it provides important insight into the mechanism for the histone ligand recognition and the effect of posttranslational modifications (PTMs) in histones, this approach neither can address the question of histone tails accessibility in the intact nucleosome core particle (NCP) nor can it reveal how independent but linked domains engage the nucleosome.

Recent remarkable advances in cryoelectron microscopy allowed the determination of structures of nucleosome–effector complexes, in which multiple intermolecular contacts, primarily with the nucleosomal core domain, were observed^15, 16^. Several nucleosome–effector complexes were also crystallized, and all show direct contacts with the NCP core domain^17–23^. However, unlike the core domain, which is globular and rigid, histone tails are flexible and not easily amenable to crystallographic analysis. A few pioneering NMR studies have investigated interactions of histone-recognizing domains (readers) with the nucleosome, containing PTMs in histone tails^24–27^, and the histone tail dynamics^28–30^. Although these studies began shedding light on the nucleosome-reader assembly in solution, NMR suffers from its own limitations. Because of the large size of the nucleosome (over 200 kDa), it remains challenging to explore the NCP-reader complexes using conventional NMR methods, and up to now, studies have been limited to characterizing the binding of a single reader.

Here we provide mechanistic analysis for the bivalent engagement of a pair of readers – the tandem PHD fingers of the CHD4 ATPase, with the intact nucleosome and demonstrate the impact of this engagement on the nucleosome dynamics. We show that the second PHD finger of CHD4 initiates recruitment to the nucleosome; however, both PHDs are required to alter the NCP dynamics. Our findings underscore the critical role of intra-nucleosomal interactions and the accessibility of histone tails within NCP for binding of readers and illuminate the importance of the nucleosomal DNA and the linker between the PHD fingers in modulating their histone-binding activities.

## Results

### PHD2 finger of CHD4 initiates recruitment to the nucleosome

**Fig. 1:**
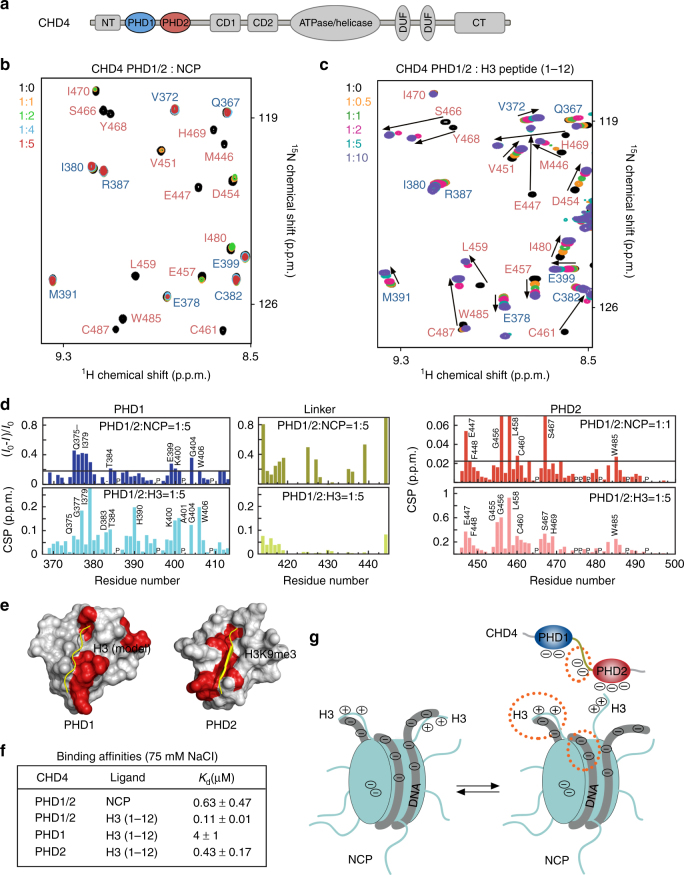
Bivalent interaction of CHD4 PHD1/2 with the nucleosome. **a** Domain architecture of CHD4. **b**, **c** Superimposed ^1^H,^15^N TROSY spectra of CHD4 PHD1/2 collected upon titration with (**b**) nucleosome or (**c**) histone H3 peptide (aa 1–12). Spectra are color coded according to the CHD4 PHD1/2:ligand molar ratio. Resonances of PHD1 and PHD2 are labeled in blue and red, respectively. **d** The bar-plots show fractional changes in intensity and CSPs in ^1^H,^15^N TROSY spectra of PHD1/2 induced by the indicated molar ratios of NCP and H3 peptide. P denotes a proline residue. **e** Identification of the NCP-binding sites of PHD1/2. Residues that exhibit NCP-induced resonance perturbations (over average + SD in PHD1 and average + 2 SD in PHD2) in **d** are mapped onto the structures of the ligand-free PHD1 and H3K9me3-bound PHD2 (PDB IDs: 2L5U and 2L75). Histone H3 peptides are yellow. **f** Binding affinities of CHD4 PHD1/2 to NCP as measured by fluorescence polarization and of CHD4 PHD1/2, PHD1, and PHD2 to H3 (1–12) peptide as measured by intrinsic tryptophan fluorescence, in buffer containing 75 mM NaCl. Error is SD based on three separate experiments. **g** A model for the association of CHD4 PHD1/2 with the nucleosome. DNA is shown as a thick gray line. The negatively charged regions in NCP or CHD4 that influence the PHD1/2 engagement with the nucleosome are encircled by orange dots

The two PHD fingers of CHD4 are connected through a short (~30 aa), primarily unstructured linker (Fig. 1a). We have previously shown that these domains, either linked or individual, are capable of binding to histone H3 peptides and that the histone-binding activity of both is necessary for proper functions of NuRD^10^. To determine the molecular mechanism by which the linked PHD1/2 fingers engage with NCPs, we examined them by NMR. We reconstituted milligram quantities of mononucleosomes, using 147 bp Widom 601 DNA and purified histone octamer, and performed ^1^H,^15^N transverse relaxation optimized spectroscopy (TROSY) NMR experiments on the sample containing uniformly ^15^N-labeled CHD4 PHD1/2 and increasing amounts of NCP (Fig. 1b). Because the interaction of CHD4 PHD1/2 with NCP results in a large complex of over 220 kDa in size that is characterized by a fast relaxation rate, the direct association between the two components was assessed through disappearance of resonances and chemical shift perturbation (CSP) analysis. The experiments were performed at 310 K to increase the overall molecular tumbling rate of the complex and decrease the signal broadening.

Gradual addition of NCP caused a substantial reduction in the intensity of PHD1/2 backbone amide resonances (Fig. 1b). Many crosspeaks, particularly of PHD2, markedly broadened and moved at the PHD1/2:NCP molar ratio of 1:1 (Fig. 1b, labeled in red). The nucleosome-binding sites of PHD1/2 were determined by plotting the intensity reduction and CSPs for each residue in the PHD1/2 construct (Fig. 1d, top panels). Mapping the most perturbed residues of PHD1 and PHD2 onto the structures of the individual domains (PDB IDs 2L5U and 2L75) revealed binding interfaces that are very similar to the binding interfaces previously identified for the association of separate PHDs or linked PHDs with histone H3 peptides^10, 12^ (Fig. 1e). A comparable pattern of resonance changes in PHD1/2 upon titration of either NCP or H3 peptide (aa 1–12 of H3) (Fig. 1b, c) further confirmed that both PHDs are engaged with the nucleosome through recognition of histone H3 tails (Fig. 1d). We note that resonances of the residues that comprise the H3-binding site of PHD2 were among the most rapidly weakened signals. Ultimately, at the molar ratio of 1:4 all crosspeaks corresponding to the backbone amides of PHD2 disappeared completely. In contrast, the PHD1 resonances only began to be perturbed (Fig. 1b, labeled in blue). Collectively, these data demonstrate that both PHD fingers of CHD4 directly interact with NCP through binding to histone H3 tails and that this interaction is mediated by the initial recognition of the tail by PHD2.

### Binding of CHD4 PHD1/2 to the nucleosome is hindered

Comparison of the PHD1/2 interactions with NCP and histone H3 peptide revealed significant differences. Unexpectedly, we found that the histone-binding activity of PHD1/2 is less pronounced toward the nucleosome. The apparent dissociation constant (*K*_d_) for the CHD4 PHD1/2–NCP complex was measured by fluorescence polarization and found to be 0.63 μM (Fig. 1f and Supplementary Fig. 1a). The apparent binding of CHD4 PHD1/2 to H3 (1–12) peptide in the same buffer (75 mM salt) was ~six-fold stronger (*K*_d_ = 0.11 μM), as measured by intrinsic tryptophan fluorescence (Fig. 1f and Supplementary Fig. 1b). A similar trend was observed for individual PHD1 and PHD2 fingers. While both bound tightly to H3 peptide (*K*_d_s of 4 and 0.43 μM, respectively), their binding to NCP became too weak to be detected by fluorescence polarization (Fig. 1f and Supplementary Fig. 1c–e). In further support, comparative analysis of resonance perturbations induced by NCP or H3 peptide suggested that PHD1/2 binds ~four- to ten-fold tighter to the histone peptide than it binds to the NCP.

**Fig. 2:**
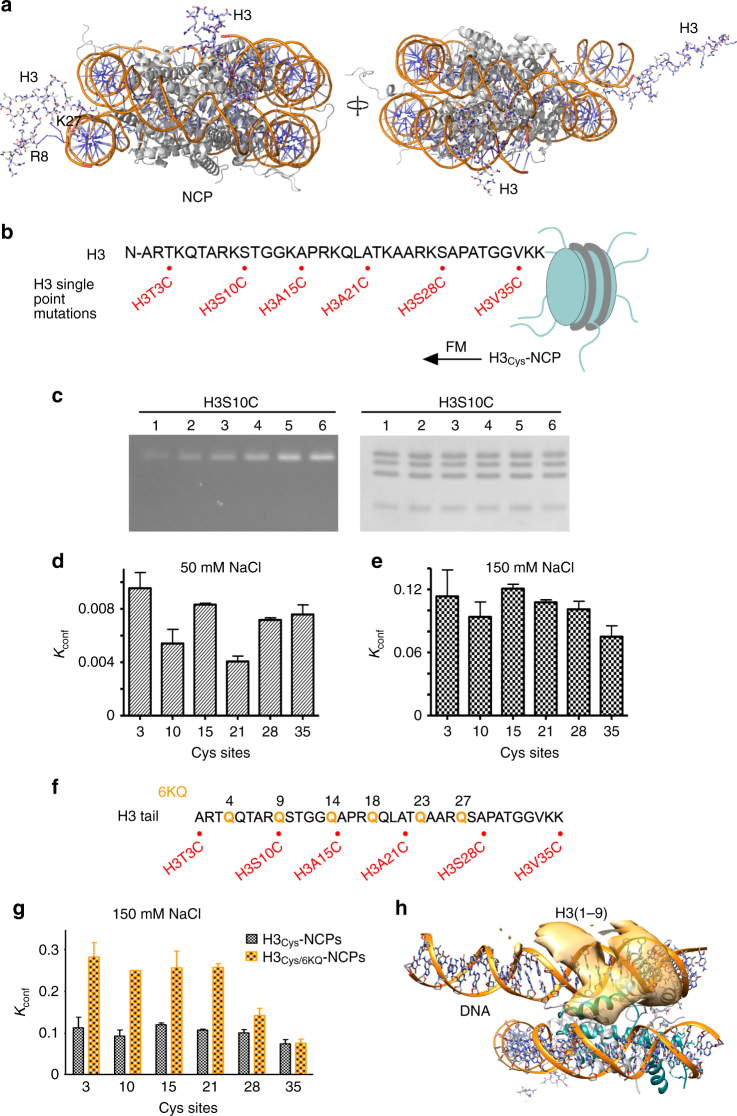
H3 tails in the nucleosome are less accessible to readers. **a** Ribbon diagram of the crystal structure of NCP (PDB ID 1KX5). Histone H3 tails are shown in a stick model with residues in close proximity to DNA labeled. **b** Schematic showing H3 tail sequence and location of cysteine substitutions. **c** Example of time course for nucleosomes containing H3S10C reacted with FM at 150 mM NaCl for 10, 20, 30, 60, 120, and 300 s shown in lanes 1–6, respectively. The gel was directly imaged by fluorography (left) then stained to reveal the histone proteins (right). **d**, **e** *K*_eq_ values determined at indicated cysteine substitutions within H3 tail in buffer containing 50 mM (**d**) and 150 mM (**e**) NaCl. The error bars represent SD based on three or more experiments. **f** Schematic showing H3 tail sequence and location of six lysine to glutamine mutations (K4Q, K9Q, K14Q, K18Q, K23Q, and K27Q) and six cysteine substitutions (T3C, S10C, A15C, A21C, S28C, and V35C) in H3 tail. **g** *K*_eq_ values determined at indicated cysteine substitutions within H3 tail. The error bars represent SD based on three or more experiments. **h** The spatial distribution of the N-terminal region (residues 1–9) of H3. The area where 30% of the tails in the ensemble are positioned is colored yellow

Why the histone-binding capability of PHD1/2 is reduced in context of the intact NCP? One possible explanation is that histone H3 tails are less accessible in the nucleosome, another is unfavorable electrostatic repulsion with nucleosomal DNA (Fig. 1g). The H3 tails protrude between two gyres of DNA near the DNA entry/exit sites and are the longest among all histone tails in the NCP. The tails are highly enriched in positively charged lysine and arginine residues that are positioned to favorably interact with the negatively charged phosphate groups of nearby located DNA. Indeed, the crystal structure of the NCP reveals transient water-mediated contacts between one of the H3 tails and DNA, while another H3 tail points away from the NCP core^31^ (Fig. 2a and Supplementary Fig. 2), and sophisticated NMR and biochemical studies show that transient contacts with linker DNA reduce H3 tail dynamics^30^.

### Intra-NCP interactions decrease the H3 tail accessibility

To experimentally define the reader-accessible regions, we investigated intra-nucleosomal binding of the H3 tails using a chemical reactivity assay. We generated six single-point mutants of H3C110A by substituting T3, S10, A15, A21, S28, or V35 individually with a cysteine residue and reconstituted NCPs containing the mutated H3s and wild-type histones H2A, H2B, and H4 (Fig. 2b). The mutation sites were chosen to evaluate the entire histone H3 tail sequence (aa 1–37 of H3) and avoid significant side-chain changes due to mutation of the histone residues to cysteine. The mutated nucleosomes and congruently mutated DNA-free H3/H4 tetramers were reacted with fluorescein-5-maleimide (FM) (Fig. 2c–e). The reaction rates were determined and conformational equilibrium constants, K_conf_, were calculated as described in methods section and ref. ^32^.

Analysis of *K*_conf_ revealed that the H3 tail within the intact H3_Cys_–NCP binds relatively tightly to the nucleosome surface at low ionic strength (50 mM NaCl) and that <1% of tails are in the NCP-unbound state and therefore available for the interaction with readers in these conditions. The *K*_conf_ values varied between 0.004 and 0.01, with the S10 and A21 sites exhibiting ~two-fold greater affinities to NCP compared to other sites tested (Fig. 2d). Interestingly, the H3S10C site is located next to the highly positively charged H3R8K9 sequence that could electrostatically contact nucleosomal DNA (Fig. 2a and Supplementary Fig. 2). Increasing the salt concentration to 150 mM resulted in an ~12-fold increase in the average K_conf_ value, corroborating primarily electrostatic nature of the interaction between H3 tails and NCP (Fig. 2e). Unlike the low salt condition measurements, there was less variation in K_conf_ values among the six sites in the elevated salt conditions, suggesting some cooperativity along the entire length of the H3 tail in binding to the nucleosome surface (Fig. 2e).

Reducing the positive charge in the H3 tail by replacing K4, K9, K14, K18, K23, and K27 with a glutamine in the H3_Cys/6KQ_-NCP led to a three-fold increase in *K*_conf_ values, however, did not abolish contacts of H3 tail with NCP, pointing to a substantial contribution not only of lysine residues but also of positively charged arginine residues to the H3 tail-nucleosome core interaction (Fig. 2f and g). Importantly, *K*_conf_ of ~ 0.1, measured for the intra-nucleosome interaction of H3 tail in near physiological ionic strength, implies that binding of readers to the H3 tails in the NCP could be reduced by up to a factor of ~10, compared to their binding to the free H3 tail peptide.

The chemical reactivity results were supported by the enhanced sampling molecular dynamics (MD) simulations data (Fig. 2h). The spatial distribution of the histone H3 tail in the conformational ensemble showed that the entire tail in NCP interacts with nucleosomal DNA. The H3 region encompassing residues 1–9, which are targeted by the PHD fingers, occupies both major and minor grooves of the outer DNA and forms transient hydrogen bonds with the phosphate groups of DNA. Collectively, MD and chemical reactivity results suggest that readers have to compete with DNA to bind to the H3 tail in the nucleosome, and that the reduced histone-binding activity of CHD4 PHD1/2 for NCP compared to its activity for the free H3 peptide is likely a result of such competition.

### The linker between PHDs increases histone binding

**Fig. 3:**
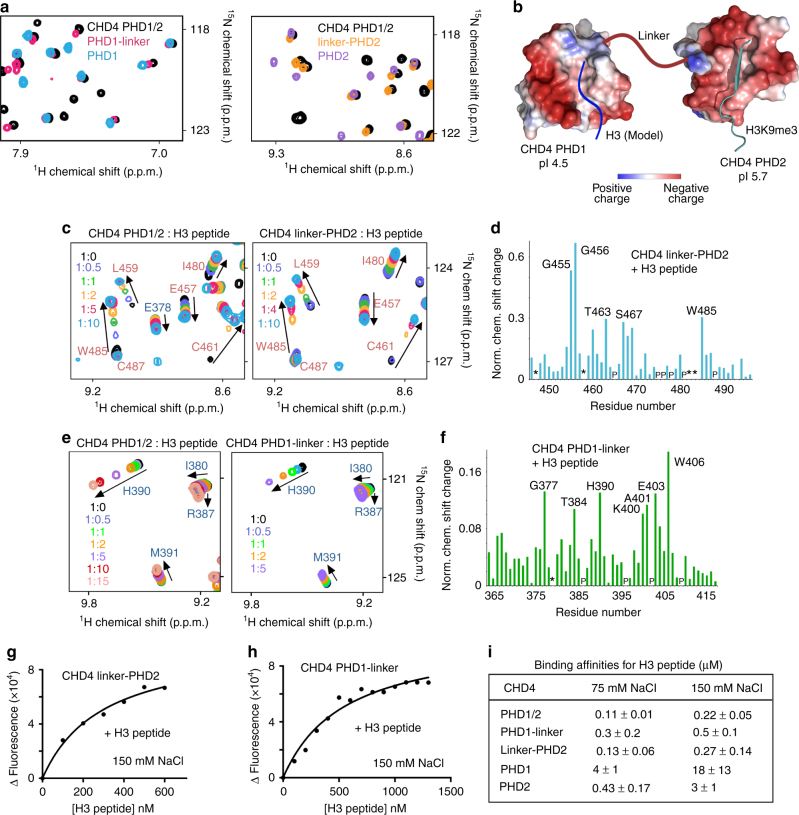
The linker affects histone binding of PHDs. **a** Superimposed ^1^H,^15^N HSQC spectra of CHD4 PHD1, PHD1-linker, and PHD1/2 (left) or CHD4 PHD2, linker-PHD2, and PHD1/2 (right). **b** The inter-domain organization of CHD4 PHD1/2. Electrostatic surface potential of PHD1 and PHD2 in complex with H3K9me3 peptide (PDB IDs: 2L5U and 2L75). Blue and red colors represent positive and negative charges, respectively. **c** Superimposed ^1^H,^15^N HSQC spectra of PHD1/2 or linker-PHD2 collected upon titration with histone H3 (1–12) peptide. **d** The histogram shows normalized ^1^H,^15^N chemical shift changes induced by the peptide in linker-PHD2. **e** Superimposed ^1^H,^15^N HSQC spectra of either PHD1/2 or PHD1-linker collected upon titration with histone H3 (1–12) peptide. **f** The histogram shows normalized ^1^H,^15^N chemical shift changes in backbone amides of the PHD1-linker due to binding to H3 peptide. **g**, **h** Representative binding curves used to determine the *K*_d_ values for CHD4 linker-PHD2 and PHD1-linker by tryptophan fluorescence. Three (PHD1-linker) or four (linker-PHD2) separate runs were performed to obtain SD. **i** Binding affinities of CHD4 PHDs to H3 (1–12) peptide as measured by fluorescence spectroscopy in buffer containing 75 mM NaCl or 150 mM NaCl. Error is SD based on three or four separate experiments

**Fig. 4:**
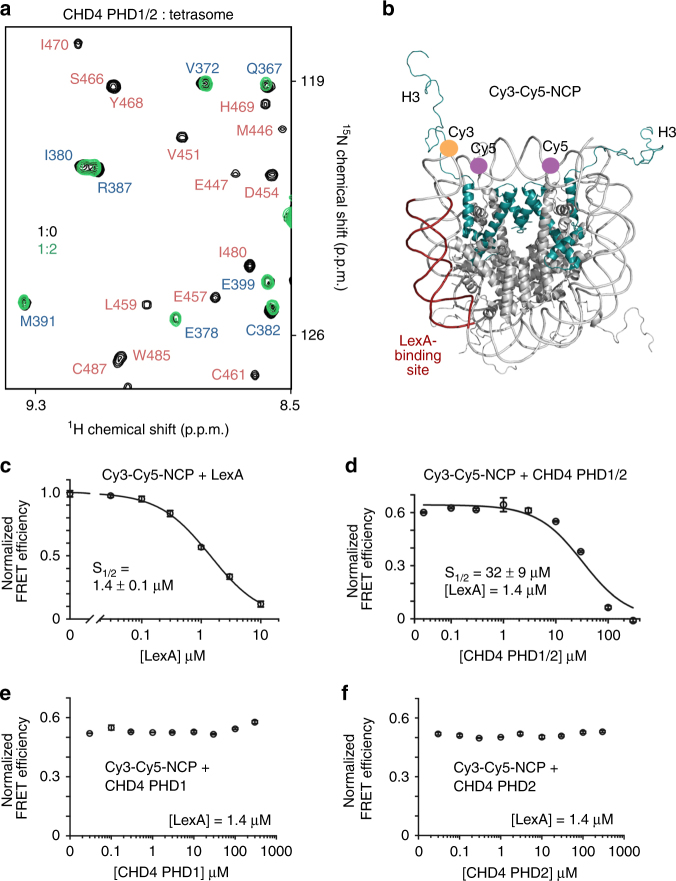
The effect of DNA and the impact on NCP dynamics. **a** Superimposed ^1^H,^15^N HSQC spectra of CHD4 PHD1/2 recorded with and without tetrasome. **b** Crystal structure of the NCP (PDB ID: 1KX5) with H3 (cyan ribbon), Cy5 at H2AK119C (purple circle), Cy3 at the 5 prime end of 601 L (orange circle), and the LexA target site (brick red) are shown and labeled. **c** FRET efficiency (E) of the Cy3-Cy5 labeled nucleosomes with increasing concentration of LexA (0–10 μM). LexA binds to its target site and traps the nucleosome in a partially unwrapped state, resulting in a decrease in FRET as [LexA] increases. The LexA titration data were fit to a binding isotherm with an *S*_1/2_ = 1.4 μM. (**d**–**f**) FRET efficiencies of the Cy3-Cy5-NCP upon titration with CHD4 PHD1/2, PHD1 or PHD2 in the presence of 1.4 μM of LexA. Error bars represent SD based on three experiments

A number of residues in the linker connecting PHD1 and PHD2 were perturbed in NMR titration experiments with either H3 peptide or NCP, suggesting that it may influence histone-binding activities of PHDs (Fig. 1d, green). To examine the role of the linker, we generated and tested the PHD1-linker and linker-PHD2 constructs (Fig. 3). An overlay of the ^1^H,^15^N HSQC spectra of PHD1, PHD1-linker and PHD1/2, and separately of PHD2, linker-PHD2 and PHD1/2 showed that some of the resonances of PHD1 and PHD2 shifted in the PHD1-linker and linker-PHD2 constructs, implying that the linker may be in contact with either domain (Fig. 3a and Supplementary Fig. 3). Gradual addition of the H3 peptide to the ^15^N-labeled PHD1-linker and linker-PHD2 NMR samples resulted in the patterns of resonance changes in PHDs that were similar in direction to the patterns of resonance changes induced in these domains in the PHD1/2 construct (Fig. 3c–f). While the linker did not alter the binding modes of PHDs (Supplemrntary Fig. 4), it increased the histone-binding affinity of both domains considerably, by ~36-fold for PHD1-linker and ~11-fold for linker-PHD2, compared to the binding affinity of the corresponding domains alone in the physiological salt concentration of 150 mM (Fig. 3g–i and Supplementary Fig. 5b, c). A ~13- and ~3-fold, respectively, enhancement in binding affinities was also observed in low salt conditions (Fig. 3i and Supplementary Figs. 1c, d and 5d, e). Since the linker is highly negatively charged (pI of 3.4), it can increase the local concentration of the positively charged histone H3 peptide, enhancing binding of the PHD fingers. Interestingly, although PHD2 contains less acidic residues (pI of 5.7) than PHD1 (pI of 4.5), the negatively charged residues in PHD2 are clustered in the H3-binding pocket. This clustering combined with potential differences in binding kinetics may account for a tighter interaction of PHD2 (Fig. 3b).

### Negative effect of DNA

A large amount of DNA present in the nucleosome may affect the engagement of CHD4 PHD1/2 with histone tails. To investigate the effect of DNA on the interaction, we prepared a tetrasome particle and collected NMR spectra of ^15^N-labeled CHD4 PHD1/2 without and with the tetrasome. The tetrasome was generated using the histone H3C110-H4T71C tetramer and a truncated Widom 601 DNA sequence (bp 34–115). Unlike the NCP with ~two gyres of DNA wrapped around the histone octamer, in the tetrasome, ~one DNA gyre is wrapped around the histone tetramer, and therefore the tetrasome is often referred to as a ‘half-nucleosome”. As shown in Fig. 4a, addition of two-fold excess of the tetrasome to PHD1/2 resulted in disappearance of the PHD2 crosspeaks and shifting of the PHD1 crosspeaks. This pattern of chemical shift changes suggested that, while PHD2 binds robustly to both NCP and the tetrasome, PHD1 associates tighter with the tetrasome than with the nucleosome. Taking into account that the tetrasome and NCP contain the same pair of H3 tails, we concluded that the negatively charged DNA plays a role in the decrease of binding of CHD4 PHD1 to the nucleosome, although we cannot rule out also the influence of H2A and H2B that are present in the nucleosome but absent in the tetrasome.

### Bivalent interaction of PHD1/2 perturbs the NCP dynamics

Nucleosomes have been shown to undergo spontaneous conformational alterations, in which the entrance/exit regions of DNA slide off the histone core, providing transient access to occluded regions of DNA^33, 34^. To determine the impact of the interaction of CHD4 PHD1/2 on the DNA unwrapping and rewrapping, we generated fluorophore-containing NCP in which the Cy3 donor is attached to the 5′-end of the 147 bp Widom 601 DNA, and the Cy5 acceptor is attached to histone H2A(K119C)^27, 35^. In the fully wrapped nucleosome, Cy3 is positioned in close proximity to one of the Cy5 fluorophores, therefore substantial energy transfer occurs (Fig. 4b). Conversely, NCP unwrapping leads to a reduction in FRET efficiency. In this assay we used the transcription factor LexA as a specific DNA-binding protein and modified 601 DNA by replacing the bases 8–27 with the LexA-binding site (Fig. 4b).

Upon titration of LexA into Cy3-Cy5-NCP, we observed a substantial decrease in FRET and, in agreement with previous measurements^34, 36^, the LexA concentration at which FRET efficiency is reduced by 50% (*S*_1/2_) was found to be 1.4 μM (Fig. 4c). Subsequent titration of CHD4 PHD1/2 into the Cy3-Cy5-NCP in the presence of LexA at the concentration equal S_1/2_ reduced the FRET efficiency, implying that CHD4 PHD1/2 binding to Cy3-Cy5-NCP perturbs the nucleosome structure shifting the DNA unwrapping-rewrapping equilibrium toward the unwrapped state (Fig. 4d). Importantly, PHD1 and PHD2 alone were unable to cause the reduction in FRET efficiency, even at concentrations of 1 mM, demonstrating that binding of a single PHD finger to Cy3-Cy5-NCP is not sufficient to produce the effect. The substantial reduction in FRET efficiency in the presence of CHD4 PHD1/2 at the concentration of 32 μM indicated that both PHD fingers must be saturated with the substrate to stimulate the nucleosome unwrapping.

## Discussion

Many chromatin-associated proteins contain multiple readers that are predicted or known to bind to histones and/or DNA. Whether or not these modules are in simultaneous contact with chromatin is one of the pressing questions that the epigenetics field has begun to address. There are a number of examples of readers tightly packed against each other that are involved in multiple concomitant interactions with histone tails or histone tails and DNA^37–39^, however, very little is known as to how readers, which are far apart in protein sequence and space, engage chromatin. In this work, we elucidate mechanistic details for the recognition of the nucleosome by independent, covalently linked readers, the PHD1 and PHD2 fingers of CHD4. We found that these readers are not equally active toward NCPs and that the second PHD finger initiates recruitment to the nucleosome. Binding to histone tails is primarily electrostatically driven, and therefore accessibility of the histone tails within the nucleosome has a notable effect on binding of PHD1/2.

Chemical reactivity, MD simulations, fluorescence, and NMR results described in this study reveal intra-nucleosomal interactions between the H3 tail and DNA and a substantial reduction in binding activity of CHD4 PHD1/2 to intact NCP compared to the free H3 peptide. Altogether, these findings imply that the reader, i.e., PHD finger must compete with the nucleosome surface for binding to the H3 tail. Depending on the extent to which any particular reader-tail interaction is compatible with intra-nucleosomal contacts of the H3 tail, our data indicate that the reduction could be as high as ~10-fold in physiological salt conditions. The strength of the adjacent readers-NCP interaction can also be modulated by intrinsic nature of the linker between readers, the length and charge of which could provide a unique way to regulate both histone- and DNA-binding activities. Importantly, the FRET data reveal the significance of bivalent contacts by PHD1/2 for promoting the nucleosome unwrapping and demonstrate that both PHD1 and PHD2 are required to produce this effect.

The finding that PHD2 of CHD4 initiates binding to the nucleosome is interesting in light of the previous data, which show that deletion or mutation of PHD2 causes a stronger de-repression of the NuRD-silenced gene *mb*-*1* in B cells compared to deletion or mutation of PHD1^10^. It is possible that PHD2 initially recruits or stabilizes CHD4 at chromatin, and PHD1 engages when the concentration of nucleosomes is high and histone H3 tails are spatially closer. In this model, PHD1 may function to “read” nucleosome density helping to stabilize CHD4 at particular chromatin regions. Another possibility is that PHD1 is fully engaged during nucleosome remodeling processes in which lower amounts of DNA are present.

In conclusion, our studies uncovered a distinctive regulatory mechanism for the association of paired readers with the nucleosome that provides a fine-tuned balance between cooperative and individual activities of the readers. Along with the PHD fingers, CHD4 contains a poly(ADP-ribose)- and DNA-binding N-terminal region, a tandem of DNA-binding chromodomains (CDs), a catalytic ATPase/helicase module and a couple of domains of unknown functions^40, 41^. Small-angle X-ray scattering studies revealed multiple inter-domain contacts and a very complex functional and regulatory interplay among the individual domains in CHD4^13, 14^. For example, interaction between PHDs and CDs was found to inhibit binding of CHD4 to nucleosomes, and the linker connecting the PHD fingers was proposed to play a role in this inhibition, as it can occlude the DNA-binding site of CDs^13, 14^. Furthermore, CDs and the ATPase domain can enhance histone binding of PHDs, whereas PHDs and CDs are in turn required for proper catalytic function of CHD4^13, 14^. These observations are also in line with the reports that the ATPase activity of CHD4 is detected on the intact nucleosomes rather than on isolated DNA or histones^42^. Clearly, studies of the full-length protein will be essential to better understand the CHD4-nucleosome engagement.

## Methods

### DNA cloning and protein purification

The CHD4 PHD1/2 (aa 361–503), PHD1 (aa 362–417), PHD1-linker (aa 361–443), PHD2 (443–498), and linker-PHD2 (aa 414–503) constructs were cloned into pGex6p1. The constructs were expressed in *Escherichia coli* BL21 DE3 pLysS cells grown in Luria Broth or ^15^NH_4_Cl supplemented minimal media with 50 µM ZnCl_2_. Bacteria were collected by centrifugation after induction with IPTG (0.5–1.0 mM) and lysed by sonication. The unlabeled and ^15^N-labeled GST-fusion proteins were purified on glutathione Sepharose 4B beads, and the GST tag was cleaved with Prescission protease. The proteins were concentrated into 20 mM Tris pH 6.8, in the presence of 150 mM NaCl and 3 mM DTT.

### NMR Spectroscopy

NMR experiments were performed on Varian 900 MHz and 600 MHz spectrometers, equipped with cryoprobes at the University of Colorado School of Medicine NMR core facility. ^1^H,^15^N HSQC or ^1^H,^15^N TROSY spectra of 0.05–0.1 mM uniformly ^15^N-labeled CHD4 proteins were collected at 298 K (600 MHz) or at 310 K (900 MHz; shown in Fig. 1b, c). The binding was characterized by monitoring CSPs in the spectra while histone H3 peptide aa 1–12 (synthesized by the University of Colorado Denver Peptide Core Facility), H3/H4 tetrasomes, and nucleosomes were added stepwise. Histograms were generated through analyzing the changes in signal intensities and SCPs per residue.

### NCP preparation for NMR experiments

6xHis-tagged or untagged *H*. *sapiens* histones H2A, H2B, and H3.2 were expressed in *E. coli* BL21 DE3 RIL cells and *H*. *sapiens* histone H4 in *E. coli* BL21 DE3 pLysS, isolated from inclusion bodies and purified using Ni-NTA beads (Qiagen, 30250) or ion exchange. To reconstitute the octamer, appropriate molar ratios of all four 6xHis-tagged or untagged histones were mixed in denaturing conditions and dialyzed against 20 mM Tris-HCl pH 7.5, 2 M NaCl, 1 mM EDTA and 5 mM DTT. 6xHis-tag was cleaved off from the histones octamer overnight by adding Tobacco Etch Virus (TEV) and PreScission proteases. The octamer was further purified by size exclusion chromatography. Thirty-two copies of 147 bp 601 Widom DNA were cloned in pJ201 with EcoRV cut sites on either end^43^. The plasmid was purified and individual copies were released with EcoRV digestion. Purification away from parent plasmid was done by PEG precipitation.

The unmodified NCPs^43^ were reconstituted by mixing 601 Widom DNA and purified octamer at the appropriate molar ratio in 2 M KCl/NaCl, 10 mM Tris pH 7.5, 1 mM DTT. NCPs were reconstituted by slow desalting dialysis into the final solution 150 mM KCl/NaCl, 10 mM Tris pH 7.5, 1 mM DTT. NCPs were further purified from free DNA by sucrose gradient.

### Tetrasome preparation for NMR experiments

Histones H3C110A and H4T71C were expressed in *E. coli* Rosetta2 (DE3) pLysS cells. The histones were extracted from inclusion bodies and purified over ion exchange columns. The tetramer was refolded into 10 mM Tris pH 7.5, 1 mM EDTA, 5 mM β-mercaptoethanol and 2 M NaCl following the protocol in ref. ^43^ and further purified over a sephacryl S-100 column (without EDTA). Single-stranded 80-bp oligonucleotides corresponding to the central portion of the Widom 601 sequence (bp 34–115) were ordered from Integrated DNA Technologies and were annealed by mixing equimolar ratios, heating to 95 °C for 10 min, and cooling to room temperature. The purified tetramer was then reconstituted by slow desalting of a 1:1 mixture of DNA and tetramer.

### Fluorescence polarization

NCPs were prepared similarly to those used for FRET assays except that the nucleosomal DNA was labeled with Fluorescein instead of Cy3 and nucleosomes contained unlabeled wild-type H2A. Fluorescence polarization measurements were carried out with increasing amounts of PHD1/2, PHD1, and PHD2 of CHD4 and 5 nM nucleosomes in 15 mM Tris pH 7.5 buffer supplemented with 75 mM NaCl, 0.00625% Tween20, and 3 mM dithiothreitol. The samples were loaded into a Corning round bottom polystyrene plate and the anisotropy measurement were acquired with a Tecan infinite M1000Pro plate reader by exciting at 470 nm and measuring polarized emission at 519 nm with 5 nm excitation and emission bandwidths.

### Fluorescence spectroscopy

Spectra were recorded at 25 °C on a Fluoromax-3 spectrofluorometer (HORIBA). The samples containing 0.5–1 μM CHD4 PHD constructs in 20 mM phosphate (or 25 mM Tris) pH 7.5, 150 mM NaCl or 75 mM NaCl, 3 mM DTT buffer and progressively increasing concentrations of the histone peptides (aa 1–12 of H3) were excited at 295 nm. Emission spectra were recorded between 320 and 360 nm with a 0.5 nm step size and a 0.5 s integration time and averaged over 3 scans. The *K*_d_ values were determined using a nonlinear least-squares analysis and the equations:

1 $${\mathrm{\Delta }}I = {\mathrm{\Delta }}{\it{I}}_{\mathrm{max}}\frac{{\left( {\left( {[L] + [P] + K_{\mathrm{d}}} \right) - \sqrt {\left( {[L] + [P] + K_{\mathrm{d}}} \right)^2 - \left. {4[P]\,[L]} \right)} } \right)}}{{2[P]}},$$

where [*L*] is the concentration of the histone peptide, [*P*] is the concentration of the protein, Δ*I* is the observed change of signal intensity, and Δ*I*_max_ is the difference in signal intensity of the free and bound states of the protein; or Δ*I* = Δ*I*_max_[*L*]/(*K*_d_+[*L*]) (2). The *K*_d_ values were averaged over three or four separate experiments, with error calculated as SD between the runs.

### Chemical reactivity assay

Recombinant wild-type *Xenopus* H2A, H2B, H4 and H3 (C110A), with cysteine substitutions and K to Q mutations in the H3 tail as indicated in Figs. 2b, 3a were expressed in *E*. *coli* BL21(DE3) cells and purified by chromatographic methods. NCPs were generated by reconstituting appropriate ratios of core histones with calf thymus DNA, followed by digestion with micrococcal nuclease to NCPs, and purification via glycerol gradients^32, 44^. Purified NCPs were incubated in 10 mM DTT for 2 h at 25 °C to ensure reduction of thiols, and after removing DTT by exchanging buffer to 10 mM Tris-HCl, pH 8.0, 10% glycerol, were stored at −80 °C. Samples of 0.8 μM NCPs, containing indicated concentrations of NaCl, were reacted with 5.6 μM fluorescein maleimide (FM), and the reactions were stopped by adding DTT to a final concentration of 5 mM. Reactions of free H3/H4 tetramers with FM were performed using quenched-flow (Kintec) by mixing equal volumes of dimers (1.6 µM) and FM (11.2 µM) for various times, then quenching with 4 volumes of 10 mM DTT^32^. The extent of FM conjugation within NCPs was performed on the benchtop by mixing NCPs (0.8 µM) with FM for different times, then quenching the reaction by adding DTT. The extent of FM conjugation was analyzed by running samples on 15% SDS-PAGE gels and quantifying band intensities by fluorography on a Typhoon (GE Healthcare) using ImageQuant. Reaction rate constants and global fits were determined using the standard single phase exponential equation, At=A0(1-e^-kt^) in GraphPad Prism. K_conf_ was calculated from the ratio of free protein and nucleosome reaction rates as determined from the plots.

### Molecular simulations

The molecular dynamics simulations in the presence of NCP were carried out using an adaptive lambda square dynamics (ALSD) enhanced sampling method^45, 46^. The initial structure of a nucleosome with 10 bp linker was modeled using NCP structures (PDB IDs 1KX5 and 1ZBB)^31, 47^. In the calculation, only atoms within a sphere of 54 Å radius at the root of the H3 tail (a nitrogen atom in the 40th residue) were considered. The simulations were carried out with an explicit solvent model at a salt concentration of 150 mM. The force fields used are an AMBER-based hybrid force field (parm94 and parm96 with a ratio of 3:7)^48^, AMBER bsc0^49^, TIP3P^50^, and Joung-Cheatham parameter^51^ for proteins, DNA, water molecules, and ions, respectively. The hybrid force field was shown to optimally reproduce the ratio of secondary structure elements determined by experimental data^48^.

To accelerate the conformational sampling, 256 independent simulations were carried out with different initial conformations. The initial conformations were prepared using a 2 ns-long MD simulation and *λ* = 0.6 (which reduces interaction energies to enhance conformational changes), starting from an extended form of histone H3 tail with different initial velocities. We performed the ALSD production run for 7.68 μs ( = 30 ns × 256 runs) in total after ALSD iterative runs to realize a random walk on the *λ*-axis^45^. The conformational ensembles obtained by production run of the same simulation length were analyzed after reweighting at *λ* = 1 based on a reweighting scheme^39^, which corresponds to conformations in the physiologically-relevant condition (300 K and 1 atm). The weight for each conformation in the ensemble indicates a relative existence probability.

### Cy3-Cy5-NCP and LexA Preparation for FRET

Nucleosomal DNA, 601 L was prepared by PCR from a plasmid containing the LexA-binding site at bases 8–27 with the Cy3-labeled oligonucleotide, Cy3-5′-CTGGAGATACTGTATGAGCATACAGTACAATTGGTC-3′ and the unlabeled oligonucleotide, 5′-ACAGGATGTATATATCTGACACGTGCCTGGAGACTA-3′. The Cy3-labeled oligonucleotide was labeled using a Cy3-NHS ester (GE Healthcare) at a 5′ amino group and purified by RP-HPLC on a 218TPTM C18 (Grace/Vydac) column. Human histones, including H3C110A^52, 53^ were expressed in *E*. *coli* and purified. Recombinant H2AK119C and H2B heterodimer was labeled with Cy5-maleimide (GE Healthcare), and Cy3-Cy5 labeled NCP was reconstituted. LexA was expressed in *E*. *coli* and separated from genomic DNA and the proteome by polyetheleneimine (Sigma) precipitation followed by salting out LexA with ammonium sulfate. LexA was resuspended in buffer B (20 mM Potassium Phosphate pH 7, 0.1 mM EDTA, 10% glycerol, 1 mM DTT) + 200 mM NaCl and purified by a linear gradient to B + 800 mM NaCl over either a cellulose phosphate or HiTrap Heparin HP Column. Final LexA purification was performed on a hydroxyapatite column and dialyzed into storage buffer.

FRET efficiency measurement experiments were carried out on a Horiba Scientific Fluoromax 4. Samples were excited at 510 and 610 nm and the photoluminescence spectra were measured from 530 to 750 nm and 630 to 750 nm for donor and acceptor excitations, respectively. Each wavelength was integrated for one second, and the excitation and emission slit width were set to 5 nm with 2 nm emission wavelength steps. FRET measurements were computed through the (ratio)_A_ method.

LexA titrations were carried out in 15 mM Tris pH 7.5, 1 mM EDTA, 75 mM NaCl, 0.00625% Tween20 with 25 nM nucleosomes. CHD4 PHD1/2, PHD1, and PHD2 titrations were carried out with a LexA concentration equal to the measured S_1/2_ of LexA binding to NCPs in the absence of CHD4 PHD1/2 (1.4 µM). Titrations were fit to *E* = *E*_0_ + (*E*_f_–*E*_0_)/(1 + (S_½_/C)) (3), where *E* is the FRET efficiency at concentration C of the titrant, *E*_0_ the efficiency in the absence of the titrant, *E*_f_ the efficiency at high titrant concentration, and S_½_ is the inflection point. Errors represent a standard deviation based on three experiments.

### Data availability

All the relevant data are available from the authors upon request.

## Electronic supplementary material

Supplementary Information
