# Supplementary material for: Accessibility of the histone H3 tail in the nucleosome for binding of paired readers
Source: Nat Commun. 2017 Nov 14;8:1489. doi: 10.1038/s41467-017-01598-x (PMC5686127; doi:10.1038/s41467-017-01598-x)
Supplement: Supplementary file 1 — Supplementary Information [file 41467_2017_1598_MOESM1_ESM.pdf]

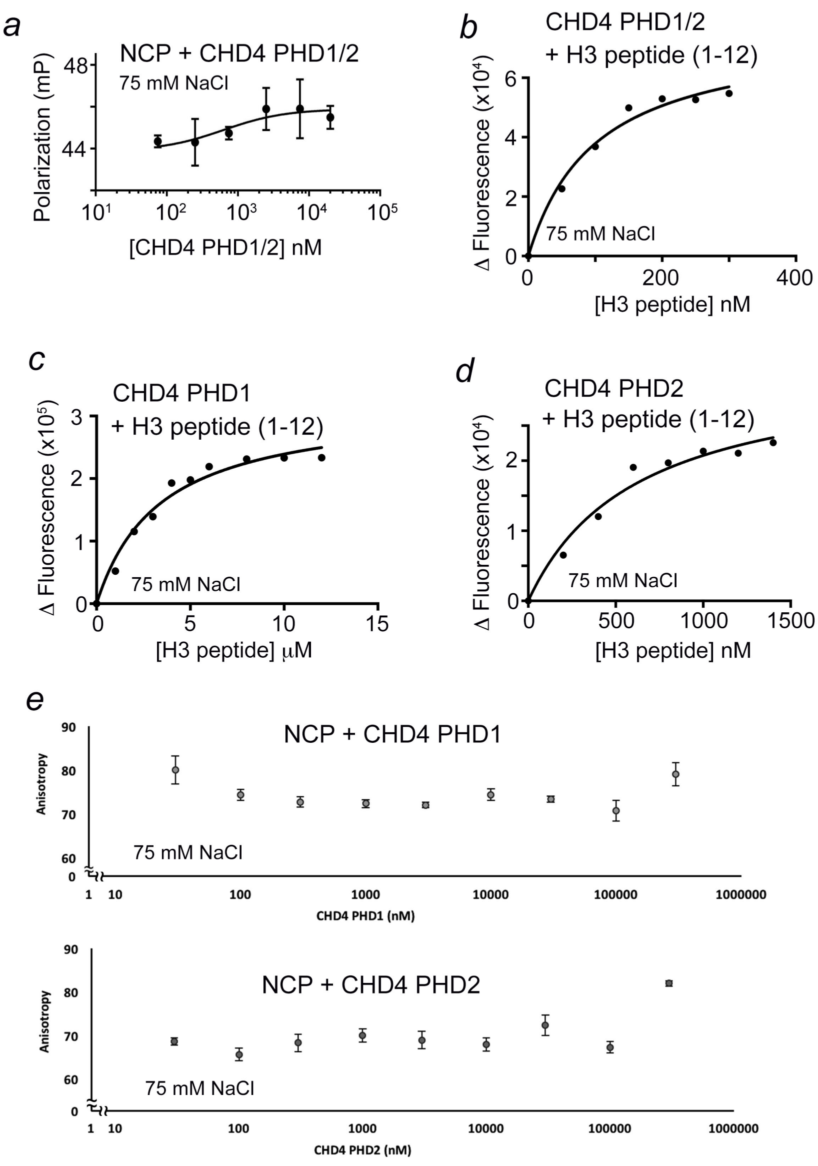

Supplementary Figure 1. (a) Binding curve used to determine affinity of the CHD4 PHD1/2 fingers to NCP by fluorescence polarization. Error bars represent a standard deviation based on three separate experiments. (b-d) Representative binding curves used to determine the K_d values for indicated CHD4 PHDs by intrinsic tryptophan fluorescence. Three or four separate runs were performed for each experiment to obtain SD. (c) Unlinked PHD fingers (PHD1 alone and PHD2 alone) are unable to induce measurable changes in fluorescence polarization experiments.

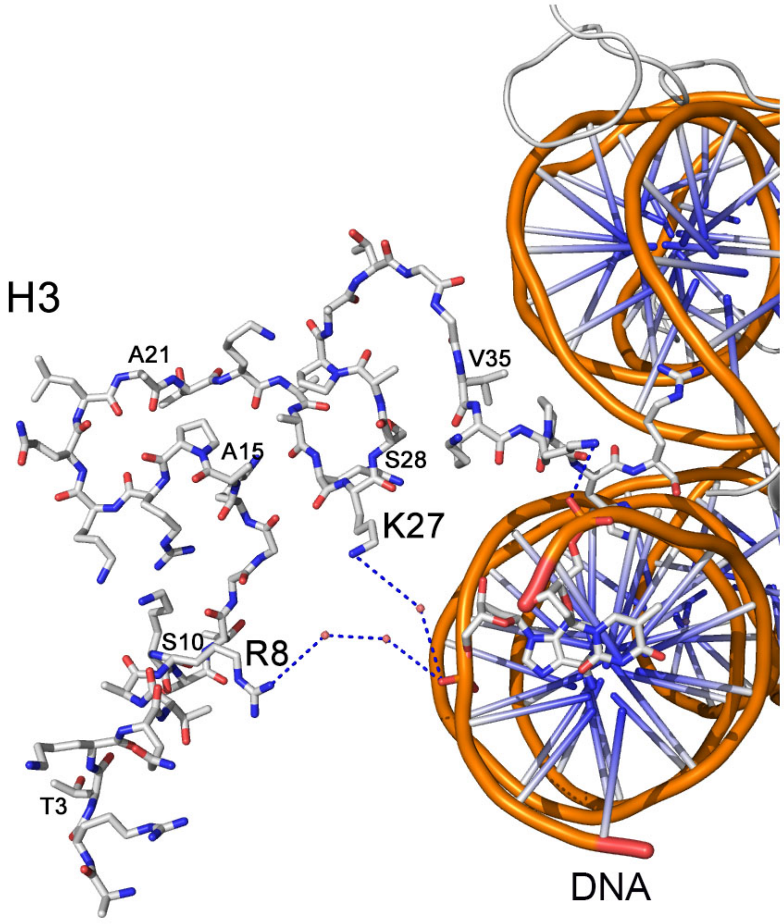

Supplementary Figure 2. A zoom-in view of the crystal structure of NCP (PDB ID 1KX5), shown in Figure 2a. Histone H3 tail is depicted in a stick model with residues in close proximity to DNA or mutated in Fig. 2b labeled. Water molecules are shown as pink spheres.

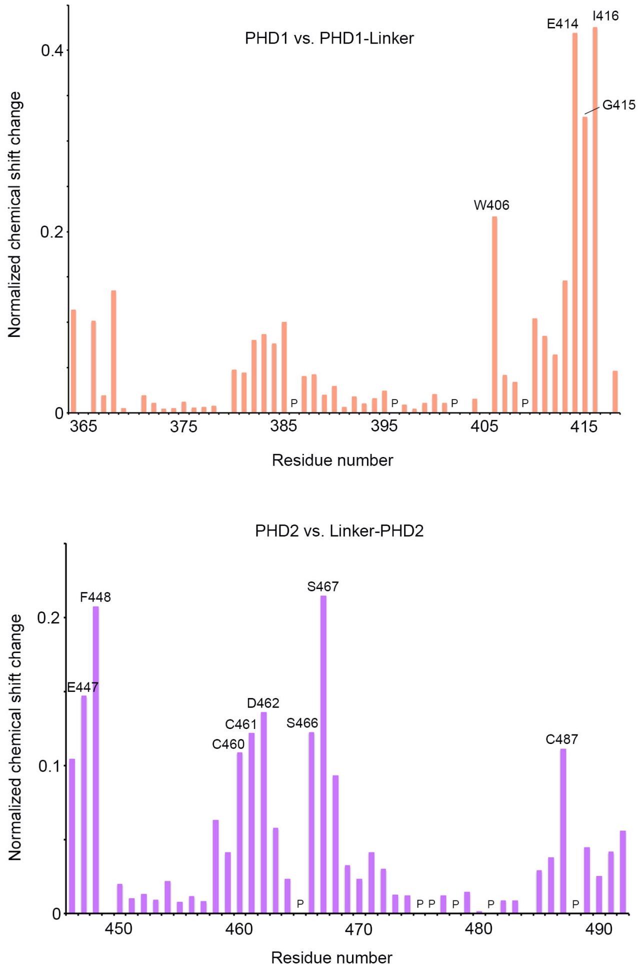

Supplementary Figure 3. The histograms show normalized $^1H,^{15}N$ chemical shift changes between the *apo* states of CHD4 PHD1 and PHD1-linker (top) and the *apo* states of CHD4 PHD2 and linker-PHD2 (bottom). Residues that show differences that are over 1 (top) and 1.5 (bottom) SD from the normalized average are labeled.

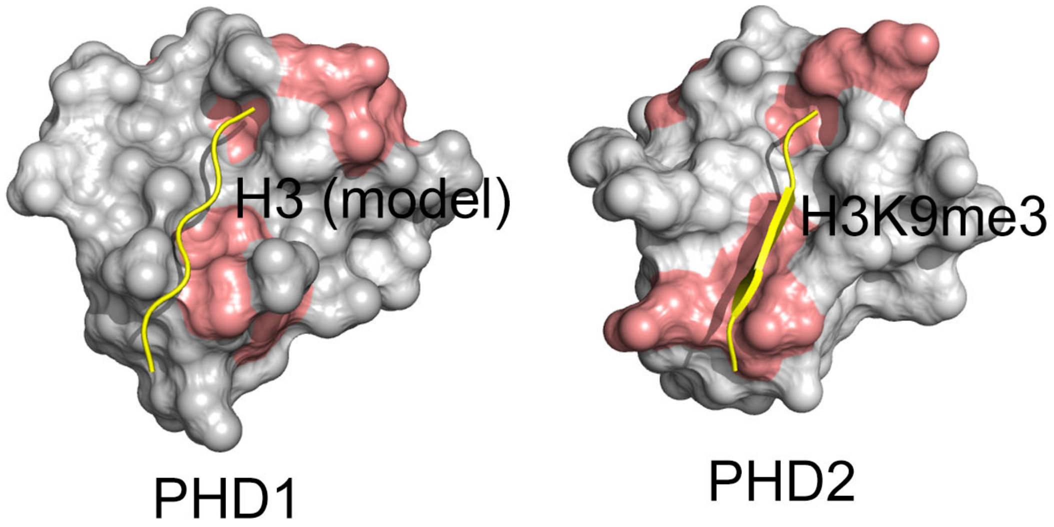

Supplementary Figure 4. Residues that exhibit H3 peptide-induced resonance perturbations (labeled in Figs. 3d and f) are mapped onto the structures of the ligand-free PHD1 and H3K9me3-bound PHD2 (PDB IDs: 2L5U and 2L75). Histone H3 peptides are yellow.

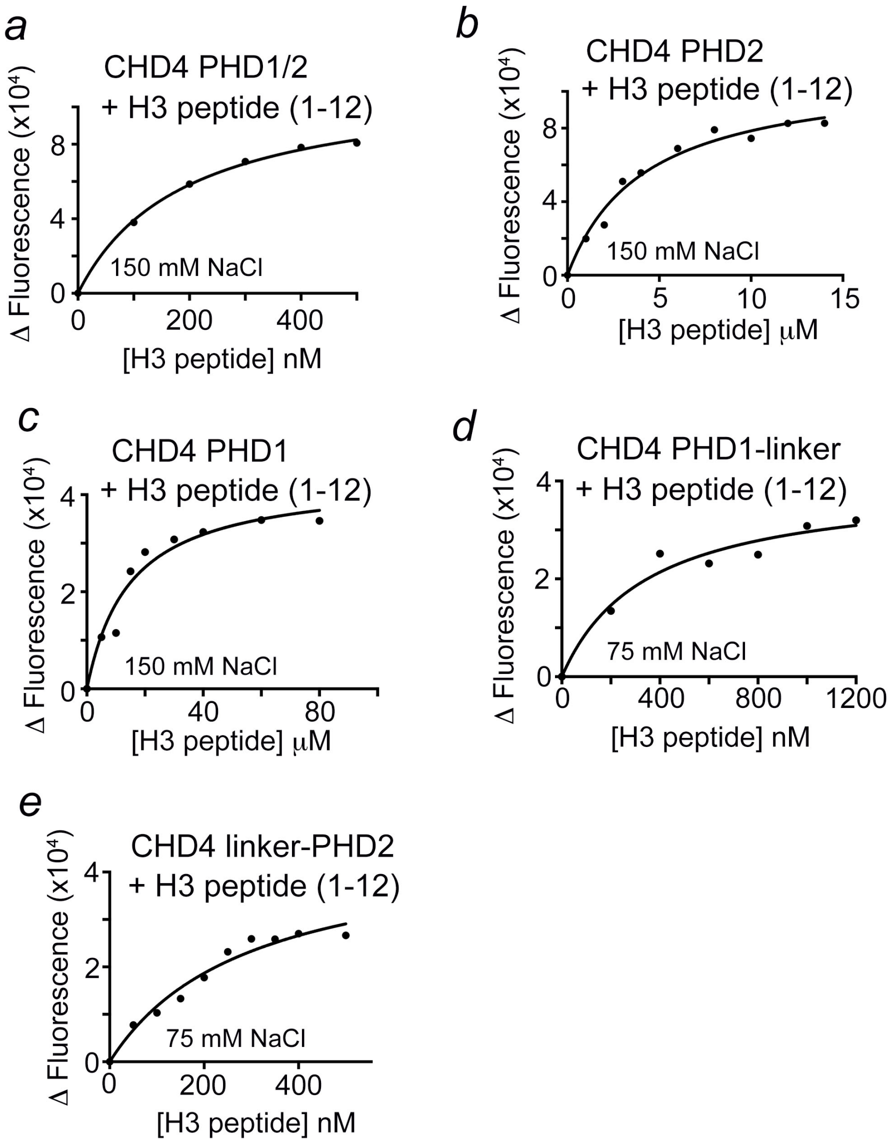

Supplementary Figure 5. Representative binding curves used to determine the K_d values for indicated CHD4 PHDs by intrinsic tryptophan fluorescence. Three or four separate runs were performed to obtain SD.
